# A rapid *Agrobacterium rhizogenes*-mediated transient expression for assessing sgRNA efficiency in CRISPR-Act3.0 in tomato

**DOI:** 10.1007/s00299-026-03792-y

**Published:** 2026-04-08

**Authors:** Karam Mostafa, Aurelia Scarano, Mohamed Farah Abdulla, Safa Hacıkamiloğlu, Orhan Kurt, Angelo Santino, Musa Kavas

**Affiliations:** 1 Faculty of Agriculture, Department of Agricultural Biotechnology, Ondokuz Mayis University, Samsun, 55200 Turkey; 2 Agricultural Research Center (ARC), The Central Laboratory for Date Palm Research and Development, Giza, 12619 Egypt; 3 Institute of Sciences of Food Production C.N.R., Unit of Lecce, 73100 Lecce, Italy; 4 Protium Technologies, Sharjah, United Arab Emirates; 5 Faculty of Agriculture, Department of Field Crops, Ondokuz Mayis University, Samsun, Turkey

**Keywords:** CRISPR-Act3.0, sgRNA efficiency, Transcription activation, *A. rhizogenes*, Fatty acid, Metabolic engineering

## Abstract

**Key message:**

CRISPR-Act3.0 is a robust tool for modulating fatty acid profiles in plants. We demonstrate that *Agrobacterium rhizogenes*-mediated transformation provides a rapid, cost-effective, and equipment-independent platform for validating sgRNA efficiency and metabolic outcomes within a short time.

**Abstract:**

The CRISPR-Act3.0 system offers a powerful strategy for activating endogenous gene expression in plants. However, the labor-intensive and time-consuming nature of stable transformation often hinders the rapid validation of multiple sgRNAs. In this study, we optimized a rapid *Agrobacterium rhizogenes*-mediated transient expression system in tomato to evaluate sgRNA efficiency within the CRISPR-Act3.0 framework. As a proof-of-concept, we targeted four genes involved in fatty acid biosynthesis*: SlFATA, SlFATB-01, SlFATB-02,* and *SlFATB-03*. To ensure precise control, we utilized the root-specific pSMB promoter to drive the CRISPRa components. Our results demonstrate that this system can successfully induce significant transcriptional activation and alter fatty acid compositions specifically increasing palmitic acid levels by up to 45%—within approximately 30 days. This approach bypasses the requirement for whole-plant stable transformation during the initial screening phase and prevents potential pleiotropic effects by restricting activation to root tissues. Overall, this study provides a highly efficient diagnostic pipeline for functional genomics and metabolic engineering in tomato, offering a significant advantage for rapid trait evaluation before committing to stable transgenic line production.

**Supplementary Information:**

The online version contains supplementary material available at 10.1007/s00299-026-03792-y.

## Introduction

Since its initial characterization as an RNA-guided endonuclease (Jinek et al. 2012), CRISPR-Cas9 has been rapidly adopted as a versatile tool for genome editing across a wide range of eukaryotic organisms, including various plant species (Hamdan et al. 2024; Tuncel et al. 2025). CRISPR-Cas9 has been primarily utilized to induce targeted mutations via the error-prone non-homologous end-joining (NHEJ) DNA repair pathway. The advent of the CRISPR/Cas genome-editing system has significantly advanced our ability to investigate and manipulate plant genomes (Hwarari et al. 2024). For over a decade, this technology has been employed to precisely modify plant DNA, facilitating functional studies of individual genes and biosynthetic pathways, as well as accelerating breeding efforts across a wide range of plant species, encompassing both model organisms and economically important crops (Belhaj et al. 2013; Kim et al. 2016). More recently, Cas9 nickase (Cas9n)-based base editors such as cytosine base editors (CBEs) and adenine base editors (ABEs) have emerged as powerful tools for introducing precise single-nucleotide substitutions in genomic DNA (Anzalone et al. 2020; Molla et al. 2021). Beyond traditional gene knockout, CRISPR activation (CRISPRa) has emerged as a powerful tool for the targeted upregulation of endogenous genes. Unlike standard CRISPR-Cas9, CRISPRa utilizes a catalytically inactive Cas9 (dCas9) fused to transcriptional activators. The optimized gRNA2.0 scaffold (gR2.0) which incorporates RNA–protein interactions derived from the bacteriophage MS2 system. In this modified scaffold, MS2 RNA stem–loop aptamers are inserted into the tetraloop and stem–loop regions of the structural gRNA. These MS2 aptamers enable the recruitment of the MS2 coat protein (MCP) fused to transcriptional activators such as VP64 to the target genomic locus, thereby enhancing gene activation efficiency (Lowder et al. 2018). In this context, a recently developed CRISPR-Act3.0 system offers an efficient platform for gene activation in plants by utilizing gR2.0 designed to recruit transcriptional activation domains, thereby enhancing the transcription of target genes. Briefly, the CRISPR–Act3.0 is composed of a synergistic combination of activation modules, including dCas9–VP64, gR2.0 scaffold, a 10 × GCN4 SunTag array, and the newly developed 2 × TAD activator to achieve robust transcriptional activation (Pan et al. 2021a). In contrast to conventional overexpression approaches, the CRISPR-Act3.0 system allows for the flexible and scalable simultaneous activation of multiple endogenous genes by specifically targeting their promoter regions. The scientists hypothesized that CRISPR-Act3.0 could serve as the foundation for an advanced CRISPR platform capable of concurrently mediating genome editing and gene activation, since the transcriptional activator is recruited through the gRNA scaffold rather than via Cas protein fusions, as was done in previous approaches (Breinig et al. 2019; Kiani et al. 2015). Assigning separate functional roles to Cas9 and gRNA, respectively, enables the creation of fully orthogonal systems for simultaneous genome editing and gene activation. Building on this concept, Pan et al. (2022) developed the CRISPR-Combo platform, a versatile system that enables simultaneous and efficient genome editing (via targeted mutagenesis or base editing) and transcriptional activation in plants (Pan et al. 2022).

While the CRISPR/Cas system has proven to be highly efficient for genome editing, its broader application and further optimization are hindered by several technical and biological challenges that remain to be addressed (Ali et al. 2023). The transformation and regeneration of most higher plant species remain labor-intensive, time-consuming, and often expensive. Beginning with initial efforts to introduce genome-editing reagents via conventional transformation methods, a range of innovative delivery strategies has since been established across various plant species. These approaches aim to enhance in vitro tissue culture responses, minimize stable transgene integration, enable rapid assessment of editing construct functionality and efficiency, and support the development of transformation and regeneration protocols that are independent of plant genotype (Cardi et al. 2023). Even in model species with well-established protocols, such as *Arabidopsis thaliana* (Chang et al. 1994; Lloyd et al. 1986) or *Medicago truncatula* (Trinh et al. 1998), generating stable transgenic lines suitable for functional analysis typically requires several months. Moreover, variability in transgene expression among individual transgenic plants is often due to position effects within the chromatin or gene silencing mechanisms, necessitating the generation and analysis of multiple independent lines to obtain reliable and reproducible data for a single transgene (Vaucheret et al. 1998). Recent advances have enhanced tissue culture responsiveness through various strategies, such as the use of epicotyl and upper stem (internodal) segments as explants in *Brassica napus* (Cao Chu et al. 2020), and the implementation of another culture-based systems in barley (Han et al. 2021). Furthermore, developmental regulators, including WUSCHEL (WUS2), BABY BOOM (BBM), and SHOOT MERISTEMLESS (STM) function synergistically with plant hormones during tissue culture to promote somatic embryogenesis or organogenesis (Gordon-Kamm et al. 2019).

The efficiency of genome-editing constructs can vary considerably. Moreover, when targeting multiple genes simultaneously using polycistronic transcripts containing multiple single guide RNAs (sgRNAs), the editing efficiency of each individual sgRNA is often unequal. Notably, sgRNAs located toward the end of the coding sequence exhibit reduced editing activity compared to those located earlier in the sequence (Fossi et al. 2019). Horlbeck et al. (2016) illustrated that editing efficiency is generally higher at 5' regions due to open chromatin and lower nucleosome density near TSS, facilitating Cas9-sgRNA access. In contrast, 3' regions often exhibit more compact chromatin and higher nucleosome occupancy, limiting accessibility and reducing editing efficiency (Horlbeck et al. 2016). Various systems have been established to evaluate the functionality of genome-editing constructs prior to their application in stable or transient transformation for generating edited plant tissues. These include the use of protoplasts (Liang et al. 2017), hairy root cultures (Cheng et al. 2021; Fan et al. 2020), plant cell suspension cultures (PCSCs), and transient expression assays in leaf epidermal cells via biolistic delivery (Budhagatapalli et al. 2016). For protoplast isolation, it is important to recognize that the optimal selection of source tissues and enzymatic digestion duration can differ significantly between plant species. Therefore, preliminary optimization is essential to identify the most effective conditions for each specific species (Chen et al. 2023). Additionally, protoplast isolation often requires specialized equipment that may not be available in all laboratories, which can limit its widespread application. In CRISPR-Act3.0-based systems for highly efficient multiplexed gene activation in plants, transient expression in protoplasts has been employed (Pan and Qi 2022).

Fatty acids (FAs) are essential components of cellular membranes and storage lipids, and they function as precursors for a wide range of plant-derived metabolites, including signaling compounds and protective phytochemicals (Lim et al. 2017). They also play a pivotal role in modulating key aspects of plant immune responses, including effector-triggered immunity (ETI), systemic acquired resistance (SAR), and various other defense-related signaling pathways (He et al. 2020; Walley et al. 2013). Acyl-acyl carrier protein thioesterases (*FATs*) play a critical role in terminating fatty acid chain elongation during the initiation of de novo fatty acid biosynthesis and are key regulators of carbon flux between the prokaryotic and eukaryotic lipid biosynthetic pathways in plants (Jones et al. 1995). In plants, acyl-ACP thioesterases are classified into two major types, FATA and FATB based on amino acid sequence homology and substrate specificity. FATA enzymes preferentially hydrolyze unsaturated acyl-ACPs, particularly 18:1-ACP, while FATB thioesterases exhibit a higher affinity for saturated acyl-ACPs, though they can also act on unsaturated substrates (Salas et al. 2002; Voelker et al. 1997). Previous studies have demonstrated that FATB-type thioesterases have a strong substrate preference for palmitoyl-ACP (C16:0-ACP), and their overexpression has been shown to significantly increase palmitic acid accumulation in species such as *Brassica napus* (Nam et al. 2019) and *Nicotiana benthamiana* (Liu et al. 2022) by facilitating the release of free saturated fatty acids from the plastid to the cytosol.

In this study, we optimized a rapid and cost-effective approach for evaluating sgRNA efficiency within the CRISPR-Act3.0 system by employing *Agrobacterium rhizogenes*-mediated transformation in tomato hypocotyl explants. This method provides a functional validation of the CRISPRa system in a relatively short timeframe—approximately one month—offering an equipment-independent alternative to stable transformation for preliminary screenings. Building upon our previously published findings (Bahadır et al. 2024), we utilized this optimized transient expression system to investigate the role of fatty acyl-ACP thioesterase (FAT) genes in regulating fatty acid composition. Specifically, we aimed to increase palmitic acid content by targeting *SlFATA, SlFATB-01, SlFATB-02,* and *SlFATB-03*. To achieve this, we employed multiplexed gene activation by designing two sgRNAs for each target gene, resulting in four individual constructs and a fifth multiplex construct containing all eight sgRNAs. This multiplex construct was driven by a tissue-specific promoter to ensure targeted expression within the root meristem.

## Materials and methods

### Selection of candidate genes for targeting

For the evaluation of sgRNA efficiency within the CRISPR-Act3.0 system, four target genes, *SlFATA, SlFATB-01, SlFATB-02, and SlFATB-03*, were selected. These targets allowed us to functionally validate the system using tomato explants as a rapid diagnostic tool.

### Single guide RNA design for gene activation

In the CRISPR-Act3.0 system, sgRNA directs a catalytically inactive Cas9 (dCas9), which is incapable of cleaving DNA but retains the ability to bind specific target sequences. The CRISPR-Act3.0 activation system targets promoter regions to modulate gene expression without inducing mutations. Before designing sgRNAs, it is essential to verify the promoter sequences of the four target genes. Specifically, we confirmed that the 1000 bp upstream regions from the transcription start site (TSS) (Supplementary file 1), obtained from Phytozome V14 (https://phytozome-next.jgi.doe.gov/), are identical to the corresponding promoter sequences of these genes in the tomato cultivar Bobcat (Fig. S1). Four primer pairs (Supplementary Table 1) were designed for PCR amplification and Sanger sequencing of the promoter regions of the *SlFATA*, *SlFATB-01*, *SlFATB-02*, and *SlFATB-03* genes.

**Fig. 1:**
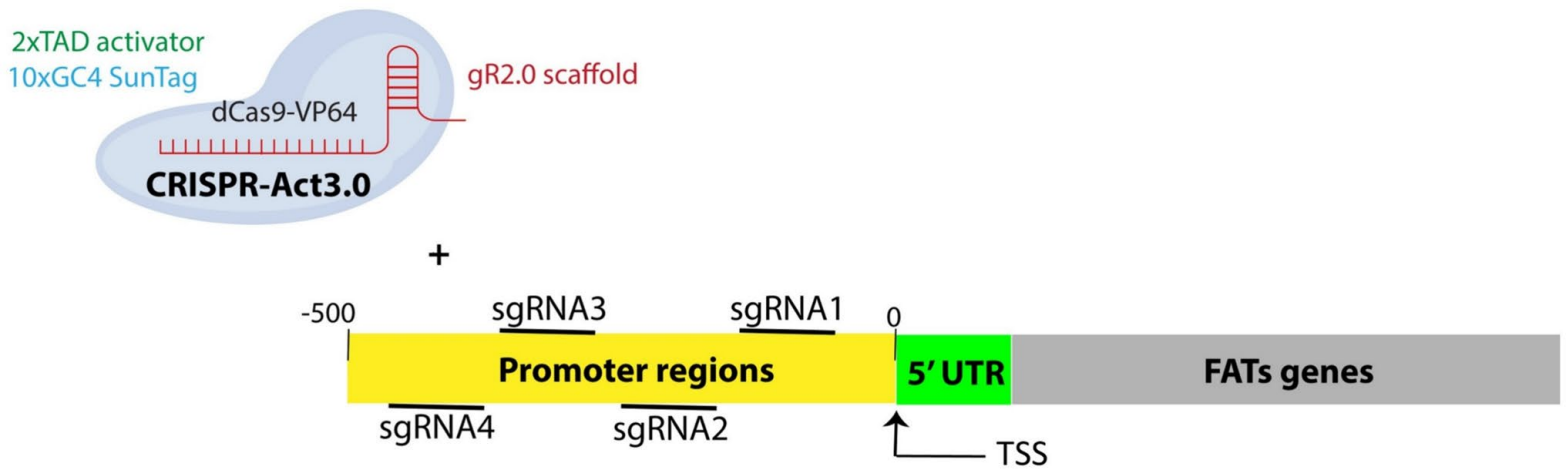
Schematic representation of a total of four sgRNAs (sgRNA1 to sgRNA4) were designed to target the region spanning from the TSS to − 500 bp upstream. sgRNAs depicted above the schematic correspond to the coding strand, whereas those shown below target the non-coding strand. UTR denotes the untranslated region; TSS indicates the transcription start site

sgRNAs were designed to enhance the expression of four target genes *SlFATA, SlFATB-01, SlFATB-02*, and *SlFATB-03*. The region containing − 5 to − 600 bp upstream of the TSS, corresponding to the promoter region, was used for sgRNAs design using the CRISPR-P 2.0 tool (http://crispr.hzau.edu.cn/CRISPR2/), and for checking the on and off-target scores using CRISPROR (https://crispor.gi.ucsc.edu/) and CHOPCHOP (https://chopchop.cbu.uib.no/) simultaneously (Fig. 1). For each gene, four sgRNAs were selected. The designed sgRNAs, ranging from 19 to 20 nucleotides in length, were synthesized as primers. Additionally, GGTC and AAAC restriction sites were added to the 5' ends of the forward and reverse sequences, respectively, to facilitate cloning (Pan and Qi 2022). Criteria for sgRNA design, including sgRNA length, GC content, the addition of 5′ nucleotide overhangs for cloning, and the incorporation of an “A” at the 5′ end when the sgRNA sequence does not naturally start with this nucleotide, were strictly followed according to the guidelines described by Pan and Qi. (2022).

### Strategic target gene identification and structural insights into sgRNA design

**Fig. 2:**
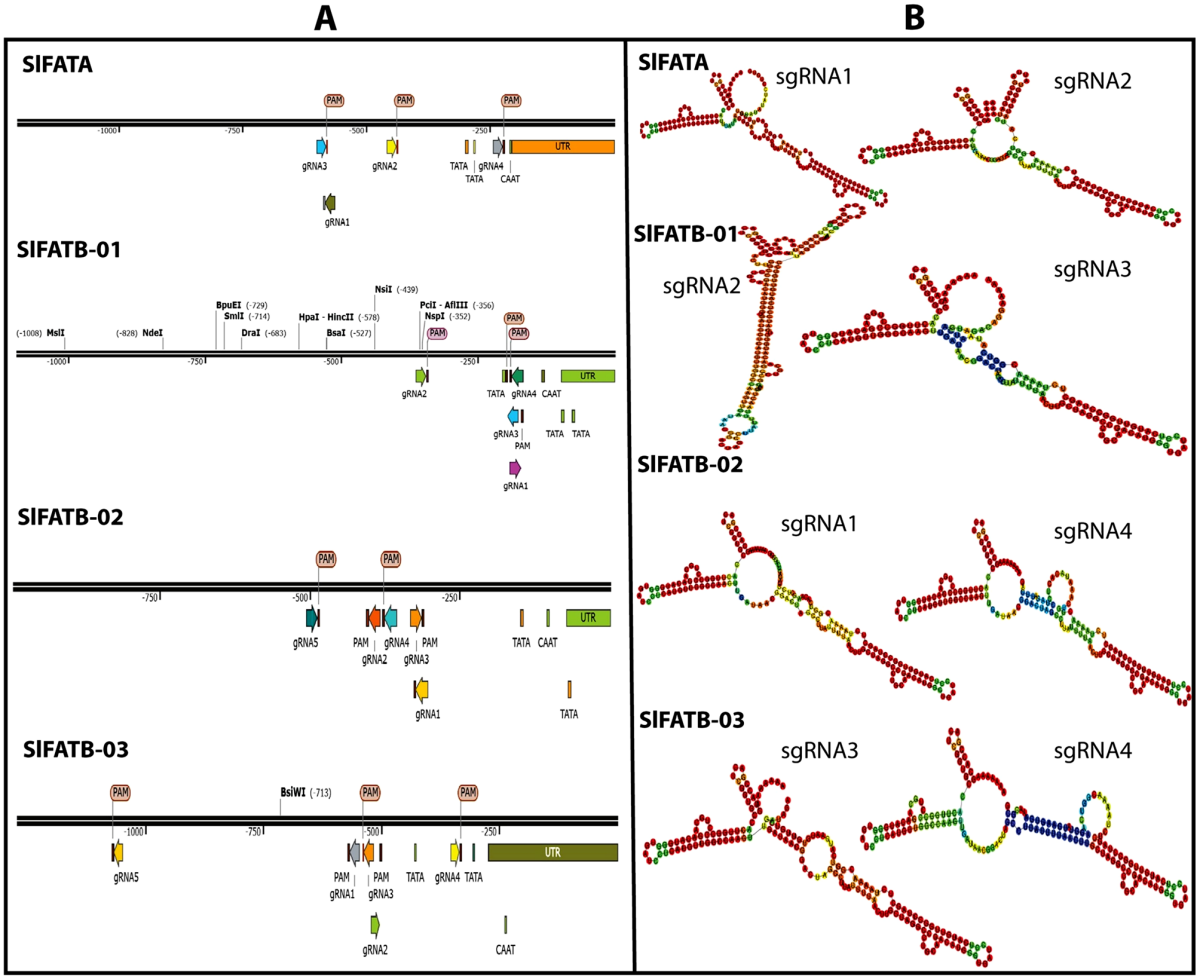
Panel A illustrates the distribution of gRNAs across the 1–500 bp upstream region of the transcription start site (TSS), highlighting their positions relative to various promoter regulatory elements such as TATA and CAAT boxes. Panel B depicts the two selected gRNAs for each gene

**Fig. 3:**
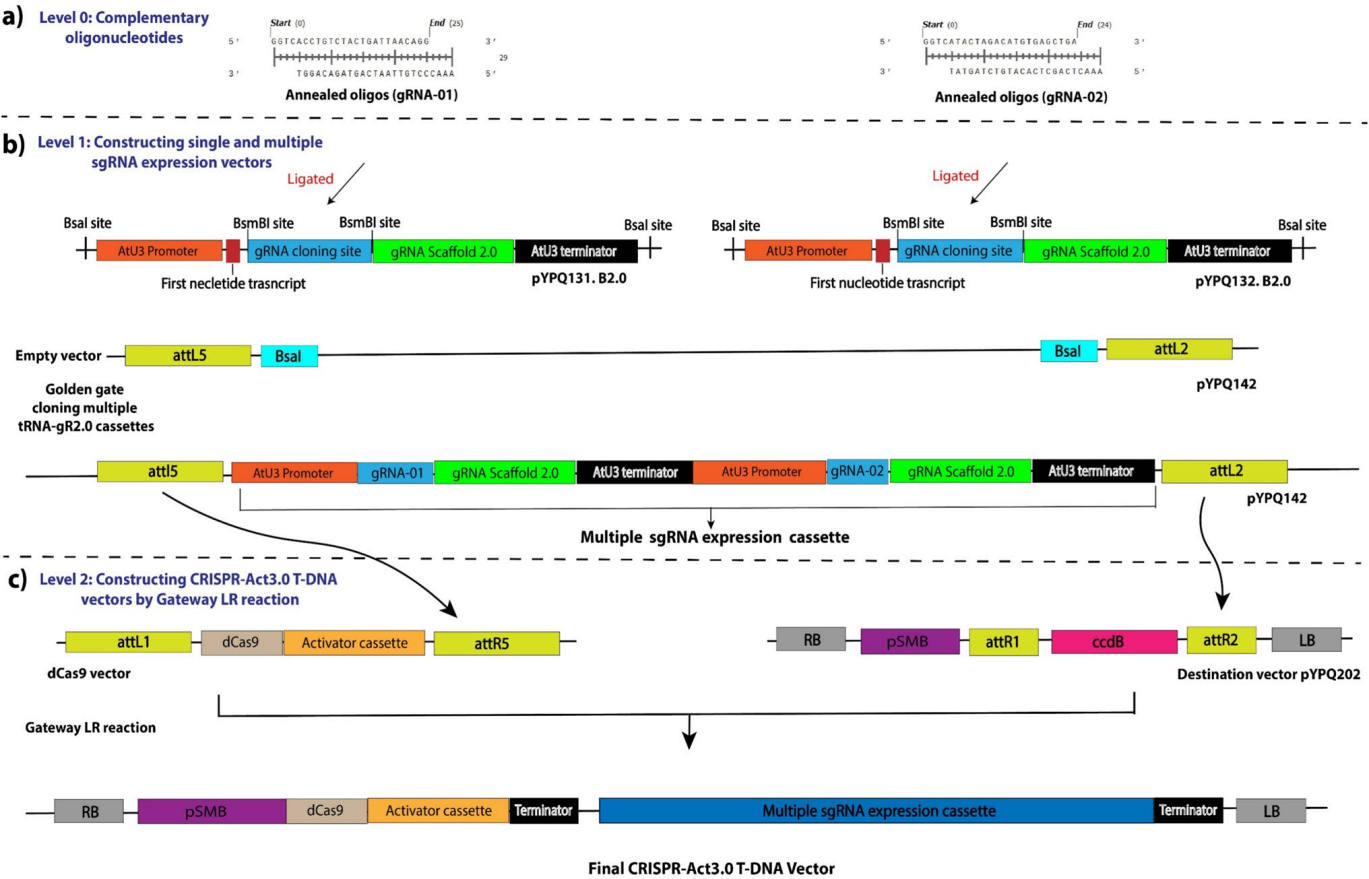
Schematic representation of the assembly of sgRNAs for multiplexed gene activation using the M-AtU3-gR2.0 system. **a** Conducted DNA oligonucleotide phosphorylation, **b** constructed single and multiplex sgRNA expression vectors, and **c** generated CRISPR-Act3.0 T-DNA vectors using the Gateway LR recombination system

Plants synthesize a wide array of fatty acid derivatives, several of which play crucial regulatory roles in modulating effector-triggered immunity, systemic resistance, and other defense-related signaling pathways. In our previous study, we systematically identified and characterized eight acyl-acyl carrier protein thioesterase (*FAT*) genes four in the *S. lycopersicum* genome and four in the *S. tuberosum* genome (Bahadır et al. 2024). In this study, we aim to achieve both individual and multiplexed overexpression of these genes employing the CRISPR-Act3.0-mediated activation approach in tomato. Four sgRNAs were designed for each gene (Supplementary Table 2), targeting regions distributed within 1–500 bp upstream of the TSS (Fig. 3 Panel A). According to the CRISPR-Act3.0 toolkit guidelines, 2–6 sgRNAs can be assembled in a single step using Golden Gate-based cloning (Pan and Qi 2022). To select the optimal two sgRNAs from the four designed for each gene, we evaluated the secondary structures of all 16 sgRNAs using the RNAfold web service (http://rna.tbi.univie.ac.at//cgi-bin/RNAWebSuite/RNAfold.cgi), as presented in Supplementary Table 2 (Hofacker 2003). The selected sgRNAs were chosen based on three criteria: predicted RNA secondary structure, minimum free energy (MFE), positional distance from the TSS, and to minimize potential overlap between sgRNAs (Fig. 2 Panel A and Supplementary Table 2); we selected two sgRNAs per gene as follows: gRNA1 and gRNA2 for the *SlFATA* gene; gRNA2 and gRNA3 for *SlFATB-01*; gRNA1 and gRNA4 for *SlFATB-02*; and gRNA3 and gRNA4 for *SlFATB-03*, as illustrated in Fig. 2 Panel B.

### Assembly of single and multiplex sgRNA expression vectors

In the original protocol, the destination vector pYPQ202 was used to perform the three-way Gateway LR recombination for expression in dicotyledonous plants. The pYPQ202 vector carries the Arabidopsis ubiquitin 10 promoter (AtUbi10) to drive expression of dzCas9 (a *Zea mays* codon-optimized version of dead Cas9). Additionally, it harbors a hygromycin resistance gene to enable selection of transgenic plants. A minor modification was introduced into the destination vector by replacing the AtUbi promoter in pYPQ202 with the tomato root meristem *S. lycopersicum pSMB* to enable root meristem-specific gene activation (Supplementary file 1). The tissue-specific promoter guides dzCas9 expression, meaning that dzCas9 will be expressed only in root tissue and will increase the expression levels of target genes specifically in the roots. The AtU3 promoter is used for each sgRNA in the polycistronic system, which means that every sgRNA has its own AtU3 promoter. The cloning system is illustrated in detail in Fig. 3.

Constructing single and multiplex sgRNA expression vectors for the CRISPR-Act3.0 system according to the M-AtU3-gR2.0 system represents one of the most challenging steps. This process involves the use of different vectors, various cloning strategies, and requires meticulous screening to identify correctly assembled constructs and successful ligations. We successfully optimized the CRISPR-Act3.0 cloning system in our laboratory. The assembly of single and multiplex sgRNA expression vectors was performed following the protocol described by Pan and Qi (2022). Briefly, sgRNA oligonucleotides (DNA oligos) were synthesized and dissolved in sterile water to a final concentration of 100 μM (Fig. 3a). Simultaneously, sgRNA entry plasmids (pYPQ131B2.0, pYPQ132B2.0, and pYPQ142B2.0, for multiplex activation) were linearized using BsmBI (Eps3I) by digesting 2 μg of plasmid DNA at 37 °C for 3 h. The digested plasmids were resolved by agarose gel electrophoresis and purified using the Favor Gel Extraction Kit. The oligos were phosphorylated with T4 PNK at 37 °C for 30 min, followed by annealing through gradual cooling from a boiling water bath to room temperature. The annealed oligos were then diluted 1:200 and ligated into the linearized sgRNA entry plasmids (pYPQ131B2.0 and pYPQ132B2.0) at room temperature for 3 h. Ligate annealed DNA oligos into linearized sgRNA entry plasmids separately (each sgRNA entry plasmids have one single gRNA). The ligation mixtures were transformed into *E. coli* DH5α competent cells via heat shock, and cells were plated on LB agar supplemented with tetracycline (10 μg/mL). Plates were incubated overnight at 37 °C (Fig. 3b).

For multiplex sgRNA assembly, two individual sgRNA cassettes (pYPQ131B2.0 and pYPQ132B2.0) were integrated into a single Gateway-compatible entry vector (pYPQ142) using Golden Gate assembly on a thermocycler (Fig. 3b). The assembly products were transformed into *E. coli* DH5α cells and plated on LB agar containing spectinomycin, IPTG, and X-gal for blue-white screening. After overnight incubation at 37 °C, white colonies were selected, and plasmids were confirmed by colony PCR. Sequence verification of the assembled sgRNA cassettes was performed via Sanger sequencing.

To generate CRISPR-Act3.0 T-DNA vectors, a three-way Gateway LR recombination reaction was carried out, assembling the dzCas9 expression vector, the multiplex sgRNA expression vector, and the destination vector pYPQ202 containing the tissue-specific promoter pSMB (Fig. 3c). Further details about the cloning system can be found in the published work by Pan and Qi 2022 (Pan & Qi 2022).

### Bacterial isolates, plant materials, and experimental growth conditions

*Escherichia coli* (DH5α) competent cells and *Agrobacterium rhizogenes* (ATCC-15834) were used in this study (Kajala et al. 2014; Ron et al. 2014a). All transformed bacterial strains were maintained in 40% glycerol stocks at − 80 °C for long-term storage. *E. coli* cultures were grown in lysogeny broth (LB) medium at 37 °C. The *Agrobacterium rhizogenes* cells were revived and cultivated in MG medium at 28 °C, supplemented with the appropriate antibiotics. In this study, the tomato cultivar ‘Bobcat’ was utilized as the plant material. Seeds were obtained from Syngenta Seed Company. Cotyledon and epicotyl tissues were used as explants for subsequent experiments.

**Fig. 4:**
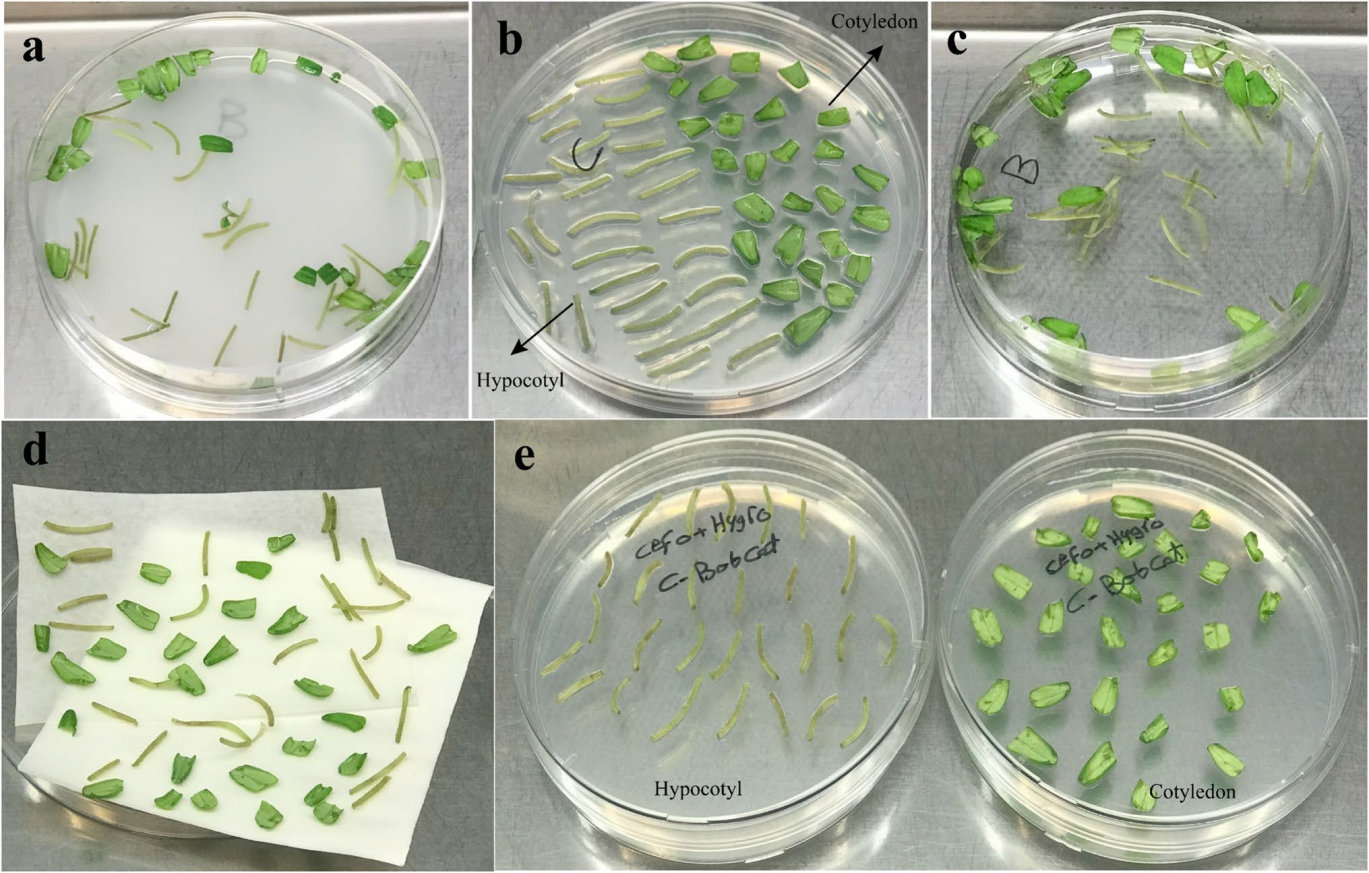
Experimental steps for *Agrobacterium rhizogenes*-based transformation protocol. **a** Cotyledon and hypocotyl explants were inoculated with *Agrobacterium rhizogenes* for 20 min. **b** Then transferred to co-cultivation MS medium (without antibiotics) and incubated at ~ 25 °C in the dark for 3–4 days. **c** Explants were rinsed twice with liquid MS medium containing cefotaxime, **d** blotted on sterile filter paper. **e** and placed onto selective root-induction medium supplemented with cefotaxime and hygromycin

Tomato seeds cv. Bobcat were surface sterilized and cultured on germination medium. *A. rhizogenes* transformation was carried out by using the SlFATA, SlFATB-01, SlFATB-02, SlFATB-03 SlFATs, and mock (Destination vector lacking any gRNA), binary destination vectors (Fig. S2). Agrobacterium-mediated transformation was applied to 10-day-old cotyledons and hypocotyl from seedlings of Bobcat cultivar. A single colony of *A. rhizogenes* was grown overnight at 28 ℃ in shaking (220 rpm) in MG/L medium supplemented with Kanamycin (50mg/L). Overnight cultures of *A. rhizogenes* were diluted to the 0.4 value at OD_600_ nm. *A. rhizogenes* cultures were centrifuged, and the pellet was resuspended in 10 mL of inoculation medium and used to inoculate cotyledon and hypocotyl explants for 20 min (Fig. 4a). Inoculated explants were transferred on co-cultivation MS media (without antibiotics) and incubated at ~ 25 ℃ in the dark (Fig. 4b), for either two or three days. After co-cultivation, the infected explants were rinsed two times with liquid MS media (containing cefotaxime) (Fig. 4c). Each inoculated explant was blotted on sterile filter paper and transferred to selective root regeneration medium (containing cefotaxime and hygromycin) (Fig. 4d and e). All explants were incubated in a plant cultivation chamber under complete darkness, with all Petri dishes covered in aluminum foil. The temperature was maintained at 24–26 °C during the day and 18 °C at night.

### Molecular verification of putative transgenic lines and quantitative real-time PCR (qRT-PCR) analysis

In order to evaluate the activation efficiency of CRISPR-Act3.0 vectors and expression of the 4 genes in the Bobcat tomato roots. Genotyping of transgenic lines was initiated by PCR amplification of the *hptII* ( +) and *VirA* (-) genes to verify successful T-DNA integration into the plant genome (Table S1). Genomic DNA was manually extracted using a modified cetyltrimethylammonium bromide (CTAB) method (Schenk et al. 2023). Root tissues were harvested from each transgenic line, immediately flash-frozen in liquid nitrogen, and stored at −80 °C until RNA extraction. Total RNA was manually isolated using a modified CTAB protocol, as described by Seçgin et al. (2020) (Seçgin et al. 2020). RNA concentration and purity were determined with a NanoDrop™ 2000/2000c spectrophotometer (Thermo, USA), and integrity was evaluated by electrophoresis on a 1.5% (w/v) agarose gel. A standardized amount of 1000 ng total RNA was used for first-strand cDNA synthesis, which was performed using the iScript cDNA Synthesis Kit (Bio-Rad, USA) according to the manufacturer’s instructions. qRT-PCR reactions were prepared with GoTaq^®^ qPCR Master Mix (Promega, USA), incorporating 1 µL of cDNA into a total reaction volume of 20 µL. Amplifications were run in triplicate on an Agilent Mx3000P system (Agilent, USA) under the following cycling conditions: an initial denaturation at 95 °C for 2 min, followed by 40 cycles of 95 °C for 15 s and 60 °C for 1 min. Relative gene expression levels were calculated using the 2^ − ΔΔCT method. The reference genes used in this study were SlUbiquitin (*Solyc01g056940*)*,* and expression levels were normalized against mock-infected controls.

### GC–MS analysis

GC–MS analysis was performed to evaluate the effects of activation genes on fatty acid composition. Root samples were oven-dried at 55 °C for 24 h. Once fully dried, the root tissues were finely ground using an electric grinder. Approximately 3 mL of petroleum ether was then added to each ground sample in a glass tube, and the mixture was thoroughly stirred with a glass rod. The tubes were sealed and left at room temperature for 30 min. After transferring the clear upper phase to new tubes, samples were placed in a desiccator overnight to evaporate the petroleum ether. The remaining oil was dissolved in 2 mL of sodium metabisulfite solution, vortexed, and incubated at room temperature for 30 min. Next, 2 mL of isooctane was added, vortexed again, and the samples were stored at 4 °C for 30 min. At the end of this period, 500 µL of the upper phase was carefully collected using a pipette and transferred to chromatography vials for analysis, following the method described by Kılınç (2011) (Kılınç 2011).

Fatty acid analysis was performed using a Shimadzu GC-2010 gas chromatograph equipped with a 20 m × 0.1 µm × 0.1 µm TR-WAX capillary column. The column oven temperature was programmed to increase to 205 °C at a rate of 6 °C min⁻^1^, with a final hold for 15 min. Hydrogen served as the carrier gas at a flow rate of 1 mL min⁻^1^. A 1 µL injection volume was used and the flame ionization detector (FID) was maintained at 280 °C. Identification and relative quantification of FA components were achieved by comparing retention times and peak areas using the Shimadzu GC Solution software, referencing a standard fatty acid methyl ester (FAME) chromatogram library.

## Results

### Establishing hairy root cultures from hypocotyl and epicotyl explants

**Fig. 5:**
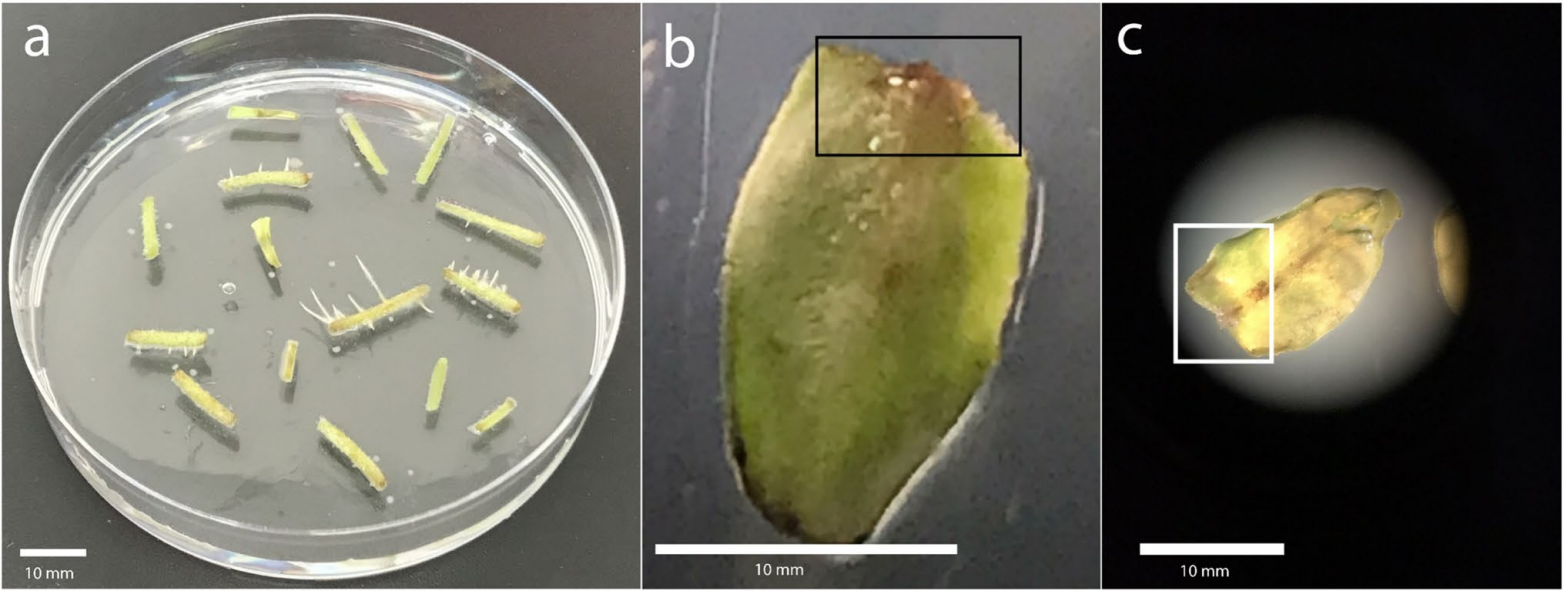
Hairy root induction in cotyledon and hypocotyl explants following *Agrobacterium* infection. **a** hypocotyls showed early swelling and root formation within one week, **b** and **c** while cotyledons developed callus with root emergence around 10 days post-inoculation. Scale bar = 10 mm

The explant types of cotyledons and hypocotyls demonstrated differential responses following *Agrobacterium*-mediated transformation. Within the first week post-inoculation, the hypocotyl explants exhibited noticeable swelling at the infection sites, which progressively developed into hairy roots (Fig. 5a). In contrast, the cotyledon explants responded by forming a light brown callus along the excised margins, with the emergence of hairy roots observed approximately 10 days after bacterial inoculation (Fig. 5b and Fig. 5c).

**Fig. 6:**
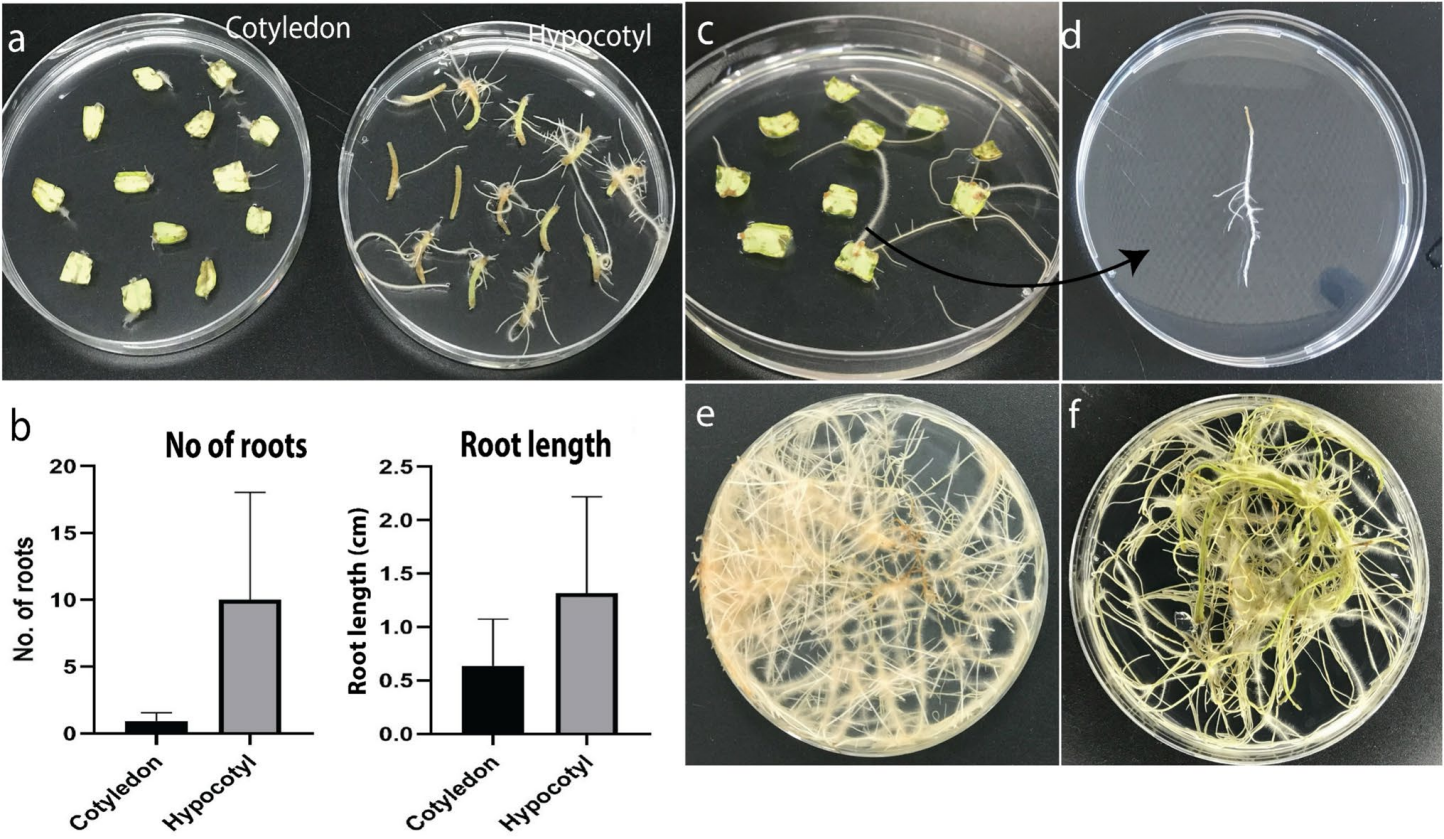
**a** Illustration of the two types of explants used: cotyledon and hypocotyl tissues after 15 days of *Agrobacterium* inoculation. **b** quantitative comparison of the number and length of roots originating from the cotyledon and hypocotyl explants. **c**, **d** selection of individual roots and their transfer to new Petri dishes, representing putative transgenic lines. **e**, **f** progression of these lines until sufficient growth was achieved for subsequent molecular analyses

After 7 days, root initiation was observed emerging from both the hypocotyl regions; after another 15 days root initiation was observed emerging from the cotyledon, however, root formation was more prominent in the hypocotyl at this stage (Fig. 6a). The number of roots originating from the hypocotyl was approximately tenfold higher compared to those emerging from the cotyledon. Additionally, the average root length from the hypocotyl-derived roots reached approximately 1.4 cm, whereas roots originating from the cotyledon averaged only 0.5 cm (Fig. 6b). These findings suggest that the hypocotyl is a more efficient and rapid site for root induction. Hairy roots arising from both cotyledonary and hypocotyl tissues were subsequently left to develop for an additional week. Each individual root was subsequently considered to represent a single putative transgenic line and was transferred to a new petri dish for further growth and analysis (Fig. 6c, d). The roots were then allowed to continue growing until they became well-developed and fully expanded (Fig. 6e, f).

### qPCR-based assessment of sgRNAs efficiency in CRISPR-Act3.0 system

**Fig. 7:**
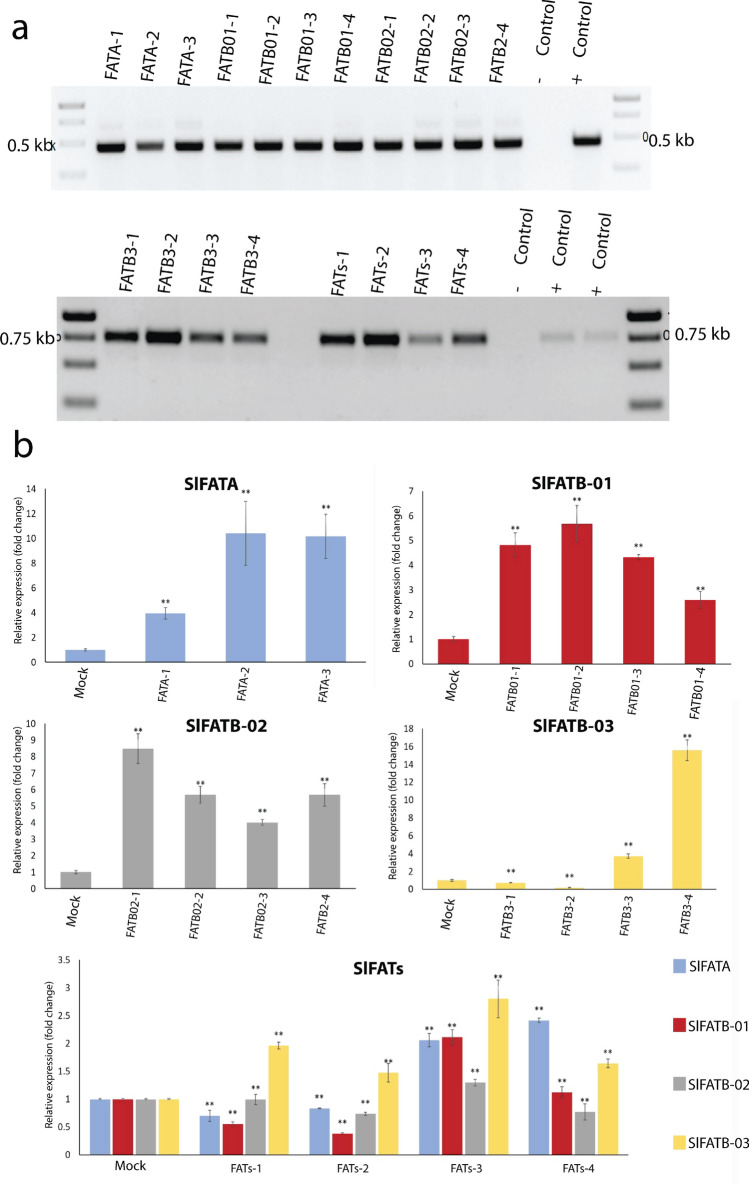
**a** Confirmation of T-DNA integration in putative transgenic lines using PCR amplification with *hptII*-specific primers. **b** qPCR analysis showing expression levels of *SlFATA, SlFATB-01, SlFATB-02, SlFATB-03*, and a combined contract set *SlFATs* designed to detect all fatty acid thioesterase genes. Mock stands for mock-infected controls. Error bars indicate standard deviations calculated from three independent biological replicates. Statistical significance was evaluated using Student’s *t* test, with *p < 0.05 and **p < 0.01 denoting significant and highly significant differences, respectively

To assess the efficacy of the CRISPR-Act3.0 vectors, gene expression analysis was performed. To verify the integration of transgenic cassettes, PCR analyses were performed employing *hptII*-specific primers as a positive control and *VirA*-specific primers as a negative control to confirm the absence of residual *Agrobacterium* contamination (Fig. 7a). To evaluate the expression levels of the targeted genes in the transgenic tomato hairy root lines, a RT-qPCR was carried out, with results presented as Log2 (2^^−ΔΔCt^) values normalized to the mock-infected controls. Both the individual gene targeting and the multiplexed genes targeting were assessed. For individual gene targeting, significant upregulation was observed. Specifically, in gene *SlFATA* (Fig. 7b), all three transgenic lines (FATA-1, FATA-2, and FATA-3) showed a significant increase in *SlFATA* expression level compared to mock-infected controls, with FATA-1 showing approximately a fivefold increase, FATA-2 reaching around 11-fold, and FATA-3 about ninefold compared to mock-infected controls. Similarly, *SlFATB-01* (Fig. 7b) showed upregulated expression levels in FATB1-1, FATB1-2, FATB1-3, and FATB1-4, in which the expression levels were increased by approximately fivefold, sixfold, 4.5-fold, and threefold, respectively. In Fig. 7b, the activation of *SlFATB-02* gene lead to a strong positive expression, with FATB2-1 showing the highest expression at roughly eightfold, followed by FATB2-2 and FATB2-4 at about fivefold, and FATB2-3 at around fourfold. Lastly, when FATB-03 was activated (Fig. 7b), the lines FATB3-1 and FATB3-2 displayed mild upregulation levels (approximately 1.5-fold each), FATB3-3 showed a stronger increase at about fivefold, and FATB3-4 reached the highest expression observed with around 16-fold over mock-infected controls.

In the multiplex experiment, where all four genes (*SlFATA-01*, *SlFATB-01*, *SlFATB-02*, and *SlFATB-03*) were simultaneously targeted using a single construct named FATs (Fig. 7b), a non-uniform expression level was observed across the different transgenic lines (FATs-1, FATs-2, FATs-3, and FATs-4). While all targeted genes generally showed higher expression than the mock-infected controls in these multiplex lines, the individual gene expression levels within each multiplex line varied. *SlFATA* expressions increased by approximately 1.6- to 2.2-fold, *SlFATB-01* by 1.4- to twofold, *SlFATB-02* by 1.5- to 2.1-fold, and *SlFATB-03* by 1.8- to threefold compared to the mock-infected controls. Overall, these qPCR results confirm the successful activation of the targeted genes in the transgenic tomato hairy roots, demonstrating the efficacy of the CRISPRAct3.0 system for both single and multiplex gene activation.

### GC–MS-based evaluation of fatty acid composition following gene activation

**Fig. 8:**
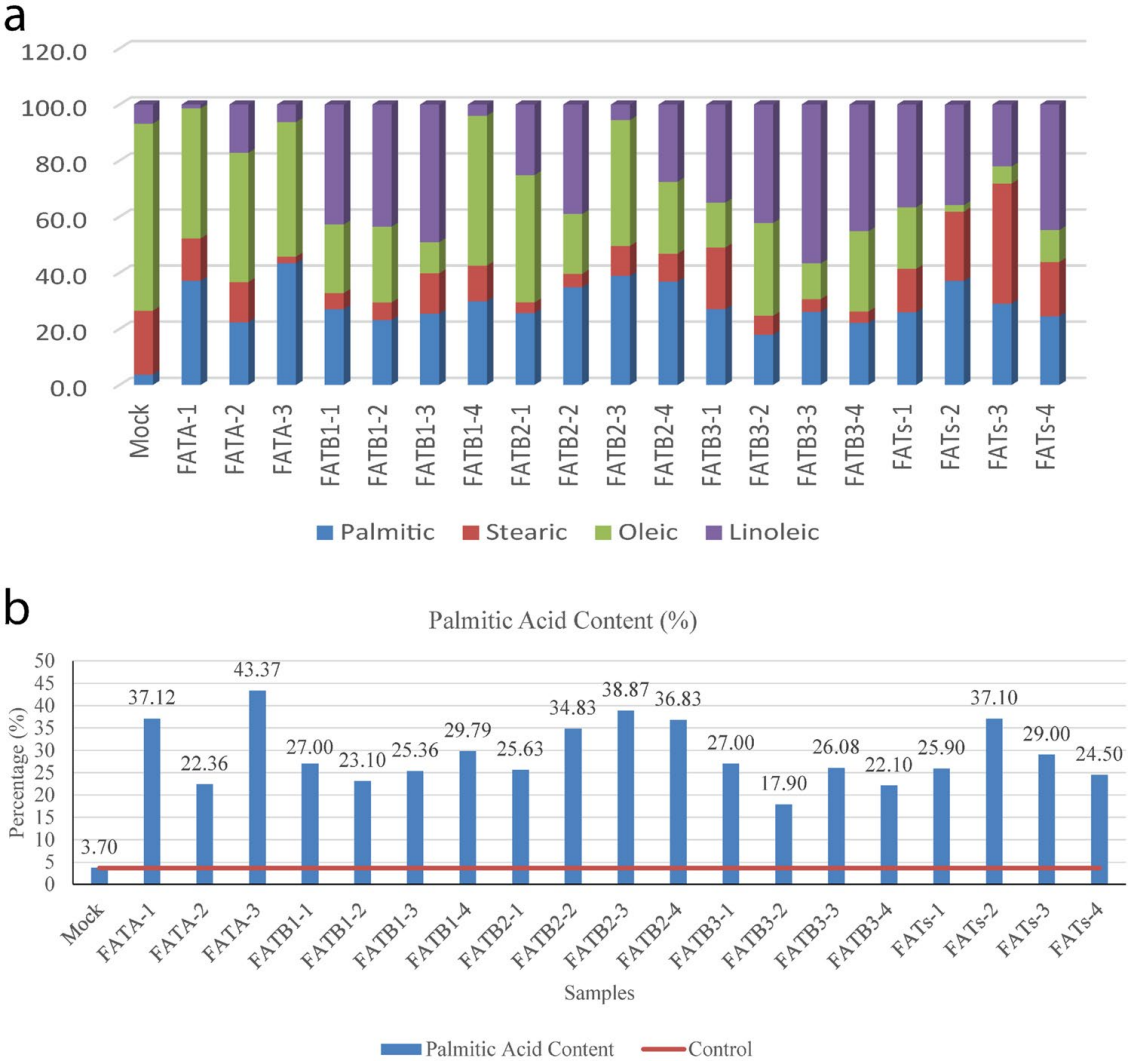
**a** fatty acid composition including palmitic acid, stearic acid, oleic acid, and linoleic acid in the putative transgenic lines. **b** percentage content of palmitic acid in these lines relative to the mock-infected controls. Mock stands for mock-infected controls

To evaluate the potential of the CRISPR-Act3.0 system in metabolic engineering aimed at modifying fatty acid composition and enhancing palmitic acid content, GC–MS analysis was performed. The results were consistent with our hypothesis, confirming the expected alterations in fatty acid profiles. According to gas chromatography results, the root fatty acid profile included palmitic acid (C16:0), stearic acid (C18:0), oleic acid (C18:1), and linoleic acid (C18:2). Palmitic acid content was elevated in all transgenic lines carrying our constructs compared to the mock-infected controls. In contrast, stearic acid levels were reduced in most lines, with the exception of FATs-2 and FATs-3, where no significant decrease was observed. Notably, oleic acid was consistently reduced across all lines relative to the mock-infected controls, suggesting a reverse relationship between palmitic and oleic acid accumulation. Additionally, linoleic acid content increased in all lines except for FATA-1, which exhibited a decrease (Fig. 8a). When focusing specifically on the percentage of palmitic acid across all transgenic lines, we observed a consistent increase compared to the mock-infected controls. Among these, line FATA-3 exhibited the highest palmitic acid content at 43.37%, whereas line FATB3-2 showed the lowest increase, reaching 17.90% (Fig. 8b).

## Discussion

Sequence-specific nucleases such as zinc finger nucleases (ZFNs), transcription activator-like effector nucleases (TALENs), and CRISPR–Cas systems have markedly advanced plant genetic research and breeding strategies (Chen et al. 2019; Schindele et al. 2020; Zhang et al. 2019). Although these tools have been predominantly utilized to generate loss-of-function mutants, functional gain studies in plants still primarily depend on traditional gene overexpression techniques. In recent years, CRISPR-mediated gene activation has emerged as a promising alternative for gene activation in plants (Li et al. 2020; Pan et al. 2021b). The CRISPRa system has evolved through multiple generations to enhance transcriptional activation efficiency. The first generation is represented by dCas9-VP64, which demonstrated modest activation capacity (Lowder et al. 2015; Piatek et al. 2015). The second generation, including systems such as dCas9–SunTag (Papikian et al. 2019), dCas9–TV (Xiong et al. 2021), and dCasEV2.1 (Chavez et al. 2015), exhibited significantly stronger transcriptional activation compared to dCas9–VP64. CRISPR–Act2.0 as another effective second-generation CRISPRa system (Lowder et al. 2018). Subsequently, Pan et al. developed a third generation of CRISPRa system, CRISPR–Act3.0, which achieved substantially higher activation potency than all prior second-generation systems.

The CRISPR system fundamentally depends on two core components: the CRISPR-associated (Cas) nuclease and a sgRNA. The selection of an appropriate nucleotide sequence (protospacer) within the target gene is a critical aspect of guide RNA design, a process that can be facilitated using specialized computational tools (Gerashchenkov et al. 2020). The emergence of computational tools in recent years has greatly facilitated the optimal design of CRISPR–Cas9 experiments. These platforms focus on improving guide RNA efficiency at the intended target site while reducing unintended off-target modifications by thoroughly evaluating target site features (Wilson et al. 2018). In our study, we utilized the CRISPR-P 2.0 online platform to design sgRNAs. CRISPR-P enables users to identify highly specific Cas9 target sites within desired DNA sequences, predict potential off-target sites for further evaluation, and conveniently annotate restriction enzyme recognition sites to facilitate downstream analyses (Lei et al. 2014). As recommended in many articles to use at least two tools for designing sgRNAs and evaluating their on- and off-target scores, we further assessed our sgRNA using both CRISPOR and CHOPCHOP (Aksoy et al. 2022). At the initial stage of our study, we designed four sgRNAs for each target gene, following the recommendation by Pan et al., who suggest designing three to five sgRNAs per gene for pre-screening in protoplast systems. Due to the limited number of suitable sgRNA target sites, we were able to design them only within the 0–500 bp region upstream of the TSS, with some overlap between sgRNAs. Following the recommendation of Pan et al., we selected two sgRNAs per gene and assembled them into a multiplexed sgRNA expression vector without prior pre-screening (Pan and Qi 2022). Two sgRNAs were selected in accordance with the findings of Liu et al.(2022), who demonstrated that transgenic rice lines harboring dual sgRNA constructs exhibited significantly higher genome-editing efficiency compared to lines expressing a single sgRNA (Liu et al. 2022). The secondary structure of the gRNA significantly influences its functional activity, with specific structural elements demonstrated to enhance guide RNA effectiveness (Doench et al. 2014; Wong et al. 2015). All selected sgRNAs validated in our experiments exhibited intact RAR stem-loop structures, along with fully preserved stem loops 2 and 3. This consistent structural conservation indicates that the integrity of these three stem-loop elements is essential for efficient genome-editing activity (Liang et al. 2016). Additionally, the minimum free energy values of all selected sgRNAs were greater than − 50 kcal/mol, a parameter that was also taken into consideration during the selection process to ensure appropriate structural stability and functionality (Supplementary Table 2) (Bruegmann et al. 2019; Hofacker 2003). Moreover, the selection of two sgRNAs in this study was performed based on the criteria outlined in the CRISPR–Act3.0 study. All selected sgRNAs were 20 nucleotides (nt) in length, consistent with previous studies indicating that, although guide RNA lengths can vary slightly, a length of 20 bp is generally recommended (Jensen et al. 2017; Pan and Qi 2022; Zheng et al. 2017). Another study reported that the spacer region of an sgRNA can range in length from 14 to 25 nt, depending on the target site and CRISPR system used (Aksoy et al. 2022). In our study, the guanine-cytosine (GC) content of all sgRNAs ranged from 35 to 60% according to Pan et al., with the exception of two sgRNAs (sgRNA2 for the *SlFATA* gene and sgRNA3 for *SlFATB-01*) that exhibited a GC % content of 30% (Pan and Qi 2022). According to Aksoy et al., a GC % content between 40 and 60% is generally preferred for the protospacer sequence, although a broader range of 20% to 80% may still be considered acceptable (Aksoy et al. 2022; Liang et al. 2016). We also considered the secondary structure of each sgRNA together with its scaffold. This approach is consistent with Aksoy et al. and Jiang et al., who describe that the stem-loop structures formed by the constant tracrRNA and crRNA regions are critical for efficient Cas9 interaction and activity (Aksoy et al. 2022; Jiang et al. 2017). In our study, the RNA polymerase III promoters, U3 small nuclear RNA (snRNA) promoter, was used to drive sgRNA expression in plants. As the U3 promoter requires a guanine-free transcription initiation site, all sgRNAs were designed to begin with an adenine (A). For any sgRNA sequences that did not naturally start with an A, an additional adenine was manually added at the 5′ end to ensure proper transcription initiation. This approach is consistent with the recommendations of Pan et al. and Hassan et al., who reported that RNA polymerase III promoters such as U3 require an adenine at the + 1 TSS to achieve efficient and accurate transcription (Hassan et al. 2021; Pan and Qi 2022).

CRISPR-mediated manipulation of essential genes often results in severe pleiotropic effects or lethality, which can limit their functional characterization in whole plants. Tissue-specific promoter systems provide an effective strategy to circumvent these constraints by restricting genetic perturbations to selected organs. In this study, we utilized the root-specific pSMB promoter (Bollier et al. 2021) to drive the CRISPR-Act3.0 system. While our current transient approach focuses on hairy root systems, the use of this specific promoter ensures that in future applications involving stably transformed plants, gene activation would be restricted to the roots. This strategic choice aims to enhance palmitic acid accumulation exclusively in root tissues, thereby preventing potential unintended metabolic or developmental effects on leaves and fruits.

The editing efficiency of CRISPR constructs can vary considerably. Moreover, when multiple genes are targeted simultaneously using polycistronic transcripts containing several sgRNAs, the individual editing efficiencies of each sgRNA are often unequal; notably, sgRNAs positioned toward the 3′ end of the transcript frequently exhibit reduced editing activity (Jacobs et al. 2017). Several techniques, such as protoplast assays, hairy root transformation, agroinfiltration, and biolistic delivery, have been employed to evaluate the efficiency of editing constructs and sgRNAs. In our study, we aimed to optimize a cost-effective, widely accessible, and equipment-independent approach for assessing the efficiency of sgRNAs from the CRISPR-Act3.0 system in tomato plants within a one-month timeframe. Hairy root cultures induced by *Agrobacterium rhizogenes* have been successfully established and utilized across various plant species for applications including biomass production, recombinant protein expression, functional genomics studies, and investigations of secondary metabolite biosynthesis (Gutierrez-Valdes et al. 2020; Ron et al. 2014b). Different strains of *A. rhizogenes* have been utilized in tomato such as *A. rhizogenes* strain A4 (Singh et al. 2014), *A. rhizogenes* strain ARqua1 (Jacobs et al. 2016), *A. rhizogenes* strain 8196 and *A. rhizogenes* strain 15834 (Lima et al. 2009). In our investigation, we employed the *A. rhizogenes* strain ATCC 15834, which is free of antibiotic resistance markers (Kajala et al. 2014).

The explant materials, cotyledon and hypocotyl, exhibited different responses following infection. Less than one week after bacterial inoculation, the infected hypocotyls displayed initial swelling, which subsequently led to hairy root formation (Fig. 5a). These findings are consistent with those reported by Lima et al. (Lima et al. 2009). The cotyledon explants exhibited distinct responses; specifically, a light brown callus formed at the cut edges, from which hairy root initiation began approximately 15 days after bacterial inoculation (Fig. 5a, b). There is a lack of research on the histological characterization of hairy root initiation from cotyledon compared to hypocotyl explants, as well as on the underlying mechanisms and differential timing of responses between these tissues. This suggests that distinct endogenous hormones, signaling molecules, and metabolic contents are likely involved in regulating these processes in each explant type.

We investigated the gene expression levels for five different constructs. Constructs 1–4 each contained two sgRNAs designed to target a single gene individually. In contrast, the fifth construct incorporated eight sgRNAs simultaneously targeting all four genes at once. The gene expression levels varied among the putative transgenic lines for the same target gene. Some lines exhibited no detectable expression, while others, such as those targeting *FATA*, showed up to a tenfold increase in FATA-2 and FATA-3 lines. In the case of the *FATB1* gene, all lines demonstrated upregulation, with fold changes of approximately 3, 4, 4.5, and 4 in lines FATB01-1, FATB01-2, FATB01-3, and FATB01-4, respectively. A similar trend was observed for the *FATB2* gene, where all putative transgenic lines exhibited upregulation. Specifically, the fold changes were approximately 4, 5, 5, and 8 in lines FATB02-3, FATB02-2, FATB02-4, and FATB02-1, respectively. For the *FATB3* gene, only two putative transgenic lines showed upregulated expression. Specifically, line FATB03-3 exhibited a threefold increase, while line FATB03-4 displayed the highest expression level, with a 16-fold increase. For the *FATs* construct containing eight sgRNAs designed for multiplex gene activation, we anticipated higher levels of overexpression compared to the other constructs. However, the highest upregulation was observed in the *FATB03* gene (2.5-fold change) across all putative transgenic lines.

In fact, we did not anticipate such relatively modest expression levels for these genes; we had expected fold changes exceeding 100, similar to what was reported by Pan et al. (Pan et al. 2021a). Notably, in their study, CRISPR–Act3.0 induced gene activation that was four to six times stronger than dCas9–TV at both target genes, achieving over 250-fold activation for *OsGW7* and over 100-fold for *OsER1*. Several factors contribute to the observed variability in gene activation efficiency among different sgRNAs targeting the same gene (Lowder et al. 2015; Papikian et al. 2019). For an accurate comparison of this system, it is essential to use the same plant species and to employ identical vector backbones and promoters to ensure consistency across experiments. In tomato, Pan and colleagues designed four distinct sgRNAs (gR1 to gR4) targeting the promoter region of the *SFT* gene. Protoplast assays revealed that gR1 and gR2 each achieved approximately 240-fold transcriptional activation, whereas gR3 and gR4 induced ~ 30-fold and ~ 20-fold activation, respectively. These results indicate that dCas9–Act3.0 exhibits strong activation potential in tomato and demonstrate that the magnitude of target gene activation is highly dependent on both the sgRNA sequence and its specific target site within the promoter region. Consequently, during sgRNA design, target sites were selected within the promoter region ranging from the TSS to − 250 base pairs upstream, as this region has been identified in prior human studies as optimal for achieving high gene activation efficiency (Chavez et al. 2015; Konermann et al. 2015). Moreover, sgRNAs designed to target the noncoding strand and possessing a GC content between 45 and 60% have been shown to achieve higher frequencies of robust gene activation (Lei et al. 2014; Pan et al. 2021a). Ultimately, the most critical factor influencing the performance of the CRISPR-Act3.0 activation system is the baseline expression level of the endogenous target gene. Comprehensive research and extensive investigation are still required to fully understand and optimize the endogenous regulatory mechanisms involved in CRISPR-based gene activation.

To investigate the functional roles of *SlFATA*, *SlFATB-01*, *SlFATB-02*, and *SlFATB-03*, we utilized the multiplexed CRISPR-Act3.0 system to activate their expression. The fatty acid composition was significantly altered in response to the enhanced overexpression of these genes. In our study, overexpression of the target genes led to an increase in palmitic acid content across the transgenic lines, reaching levels of up to 45%. This result is consistent with the findings of Tang et al., who demonstrated that in oil-rich crops like peanuts, disruption of *FAT* genes significantly modifies the fatty acid profile, notably decreasing palmitic acid concentrations while enhancing oleic acid levels (Tang et al. 2022). In *Arabidopsis*, *AtFATB* regulates saturated fatty acids, affecting membrane composition and the synthesis of key cellular components essential for plant growth (Bonaventure et al. 2003). Parallel to the results found in this study, the soybean double mutant *GmFATB1a:1b* showed reductions of 42% and 35% in palmitic and stearic acid levels, respectively, accompanied by growth abnormalities and male sterility (Ma et al. 2021). The fatty acid composition in plants is a complex phenomenon that requires further investigation. Additionally, the rapid metabolic turnover of oil quality presents a significant challenge for researchers studying changes in fatty acid profiles. Metabolic engineering in plants is more complex than anticipated. Although *FATB3-4* exhibited the highest expression level as determined by qPCR, this did not correspond to the highest palmitic acid accumulation among the tested lines. Various molecular-level factors influence the translation of specific genes, including post-translational modifications and other regulatory mechanisms (Mipeshwaree Devi et al. 2023). Moreover, in primary metabolism, regulation of biosynthetic pathways is largely governed at the genetic level; however, specific biosynthetic processes can be modulated by external stimuli, such as environmental stresses. These stimuli trigger cascades of signaling events through the production of various signaling molecules (Ashraf et al. 2018). Nevertheless, the outcomes of metabolic engineering are often unpredictable, primarily due to limited understanding of substrate availability within specific subcellular compartments of the target tissues, uncharacterized or latent biochemical activities (‘silent metabolism’) within engineered cells, and complex and unrecognized crosstalk among interconnected metabolic networks (Lynch et al. 2021). This concept highlights the existence of hidden constraints that, although not readily apparent under natural conditions, limit the effectiveness of metabolic engineering and underscore the need for future research to identify and overcome these limitations.

Although this study utilizes a hairy root system for rapid validation, the implementation of the root-specific pSMB promoter provides a strategic framework for future whole-plant research. Unlike ubiquitous promoters, the pSMB-driven CRISPR-Act3.0 ensures that gene activation remains restricted to the root system, thereby circumventing the pleiotropic effects or lethality often associated with the overexpression of essential metabolic genes in other tissues. This approach establishes a reliable pipeline where sgRNAs are first validated in a one-month timeframe before proceeding to the generation of specialized, stably transformed lines.

## Supplementary Information

Below is the link to the electronic supplementary material.

Supplementary file1 (TXT 7 KB)

Supplementary file2 (PNG 7501 KB)

Supplementary file3 (JPG 289 KB)

Supplementary file4 (XLSX 36 KB)

Supplementary file5 (XLSX 1182 KB)

## Data Availability

All data generated or analyzed during this study are included in this published article (and its Supporting Information files). The materials used in our study are available under an MTA from the corresponding author upon reasonable request.
