# Supplementary figures and images for: A rapid Agrobacterium rhizogenes-mediated transient expression for assessing sgRNA efficiency in CRISPR-Act3.0 in tomato
Source: Plant Cell Rep. 2026 Apr 8;45(5):118. doi: 10.1007/s00299-026-03792-y (PMC13061811; doi:10.1007/s00299-026-03792-y)

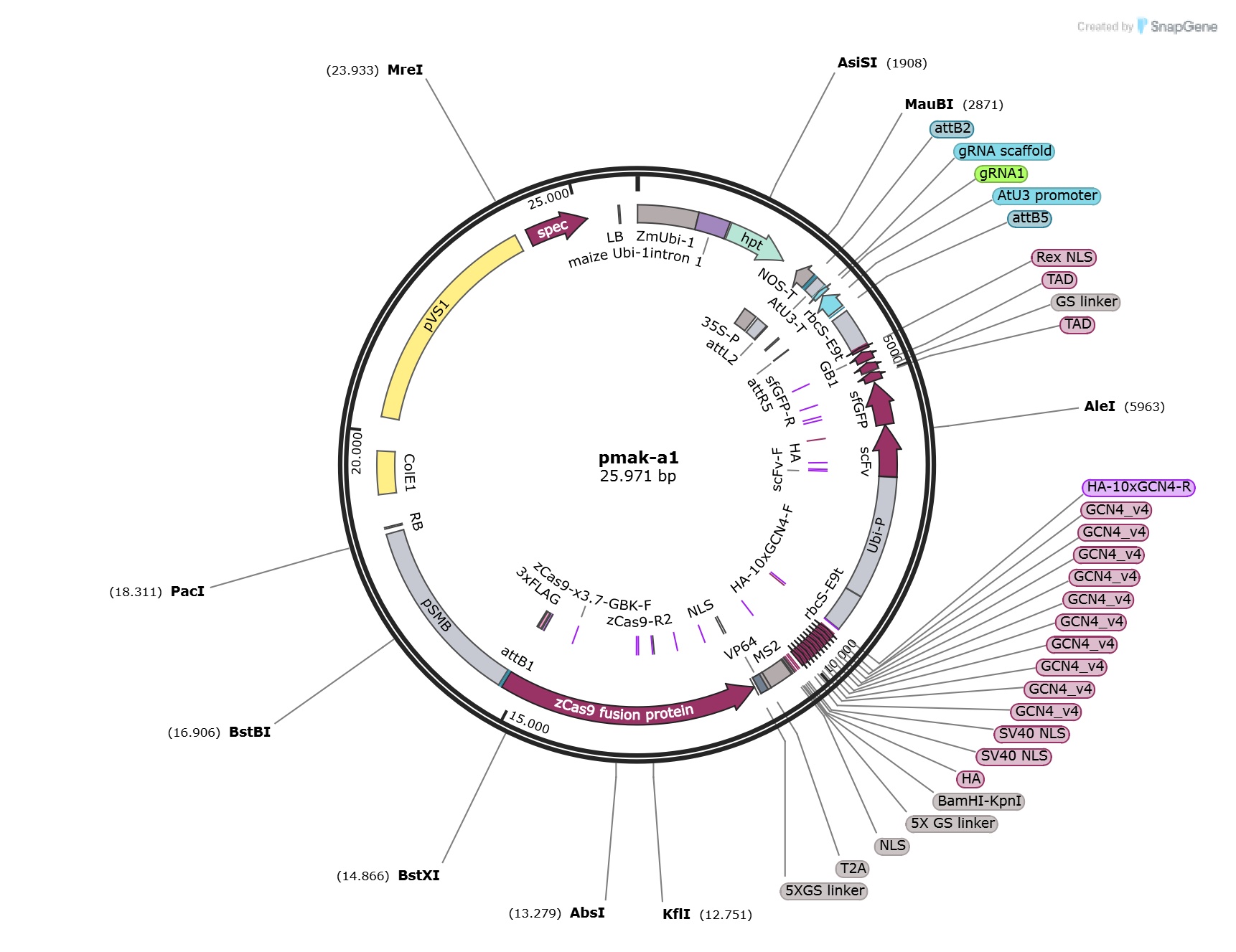

Supplement: Supplementary file 3 — Supplementary file3 (JPG 289 KB) [file 299_2026_3792_MOESM3_ESM.jpg]
